# Venous Doppler in the Evaluation of Fetal Hydrops

**DOI:** 10.1155/2010/430157

**Published:** 2010-05-05

**Authors:** C. Hofstaetter, S. Gudmundsson

**Affiliations:** ^1^Department of Obstetrics and Perinatology, University of Marburg, 30559 Hannover, Germany; ^2^Department of Obstetrics and Gynecology, Malmo University Hospital, 20502 Malmo, Sweden

## Abstract

*Objective*. To examine venous blood flow velocity in different types of fetal hydrops and its value in the prediction of outcome of pregnancies. *Methods*. Venous Doppler sonography was performed in 100 hydropic fetuses from 15 to 37 weeks of gestation. Blood velocity was recorded in the right hepatic vein (HV), the ductus venosus (DV) and in the intra-abdominal part of the umbilical vein (UV). Blood velocity indices were calculated and pulsations in the umbilical vein noted and grouped into a single, double or triple flow pattern. Blood velocity was related to cause of hydrops. *Results*. Mortality was noted in 51 cases of which 19 were by termination of pregnancy. Mortality in the 30 with normal venous blood velocity was 35%, but 58% in cases of abnormal Doppler. Abnormal HV and DV blood velocities were recorded in 39 and 34 cases, respectively and were strongly related to mortality (*P* < .04 and *P* < .003, resp.). UV pulsations were noted in 49 fetuses and were significantly related to mortality (*P* < .04). Mortality and abnormal venous velocities were most frequent in the low-output hydrops group (79% and 75%, resp.). *Conclusions*. Abnormal venous blood velocity is related to mortality in pregnancies complicated by fetal hydrops. Venous Doppler sonography should be a part of the routine work-up of pregnancies complicated by fetal hydrops.

## 1. Introduction

 Fetal hydrops is a serious condition defined as abnormal accumulation of fluid in 2 or more fetal body compartments, including asides, pleural effusion, pericardial effusion, and skin edema. In some patients, it may also be associated with polyhydramnios and placental edema. The basic mechanism for the formation of fetal hydrops is an imbalance of interstitial fluid production and the lymphatic return. Fluid accumulation in the fetus can also result from congestive heart failure, obstructed venous or lymphatic flow, and decreased plasma osmotic pressure.

 Fetal hydrops is easily detectable by ordinary ultrasound due to skin edema and fluid collections in the body cavities. The incidence is 1 : 1500 to 1 : 4000 pregnancies [1]. Secondly to fetal circulation congestion, polyhydramnios and placental hydrops may often also be detected. Perinatal mortality is 70%–90% and treatment is still difficult and limited [2], but surveillance and possible treatment require information about the pathophysiological mechanism that leads to fetal hydrops.

 Doppler ultrasound has improved our understanding of fetal circulation and the patho-physiological mechanism that controls fetal circulation. Doppler sonographic studies on fetal arteries have not given any characteristic findings that might predict the cause or outcome of cases with hydrops. 

 Blood velocity in the fetal systemic veins has a characteristic pulsating pattern as a reflection of pressure in the right cardiac atrium [3, 4]. Normal umbilical venous blood flow and flow in the portal circulation are even and without fluctuation due to the filtering effect of the ductus venosus. During fetal heart failure or hypoxia, a decrease in blood flow to the heart can be seen in diastole in the systemic veins as a reflection of increased central venous pressure [5]. Due to the opening of the ductus venosus, a pulsating pattern appears in the umbilical vein. Umbilical venous pulsations have previously been proposed to be able to differentiate between a fetus with hydrops due to heart failure and other causes such as viral infection [6]. 

 There are different pathological processes that can lead to fetal hydrops. An attempt has been made to group cases with fetal hydrops based on the following causes: high-output heart failure, low-output heart failure, hydrops caused by disturbed lymphatic flow, hydrops caused by obstructed venous return, and hydrops of idiopathic cause, which might be viral infection in some cases [7]. Specific antenatal diagnosis is important in order to predict prognosis during counseling the parents. The aim of the present study was to evaluate fetal venous blood flow in cases with hydrops in relationship to the different etiological groups and see which venous blood velocity recording could best predict outcome of pregnancy.

## 2. Methods

 One hundred hydropic fetuses were examined in a prospective study between 15 and 37 completed weeks of gestation (average of 26 ± 5 weeks). A careful ultrasound examination, including echocardiography as well as color and pulsed Doppler sonography were performed with real-time ultrasound machines by Acuson-type 128XP 10 and Aspen (Mountain View, California, USA), with 3.5 and 5.0 MHz transducers, and by Siemens-type Sonoline ELEGRA (Siemens Medical Systems Inc., Issaquah, Washington, USA), with 3.5 to 5.0 MHz transducers. The maximal ultrasound intensity was set at 100 mV/cm^2^ spatial peak temporal average and the high-frequency filter was set at 125 Hz to remove signals from slow-moving tissues.

 In B-mode ultrasound, the presence and severity were determined by skin edema and fluid collection in abdominal, pericardial, and pleural cavities. Hydrops was defined as collection of fluid in at least two fetal body compartments. The hydropic fetuses were divided into four groups according to patho-physiological conditions causing fetal hydrops (Table 1). 

Atrioventricular (AV) valves blood velocity was examined by color and pulsed Doppler ultrasound. Regurgitation was recorded and defined as early if only in the beginning of systole or holosystolic if leakage was recorded throughout systole. 

**Figure 1:**
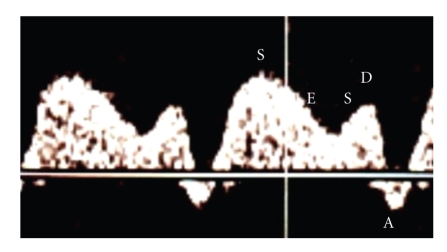
Spectrum of blood velocities in right hepatic vein: S = ventricular systole, ES = end-systole, D = peak diastolic velocity, and A = atrial contraction in late diastole.

The umbilical artery was also examined at a free-floating loop of the cord and pulsatility index (PI) was calculated using the method used by Gosling et al. [8]. Hepatic veins (HVs) were located in a transverse view between the ductus venosus (DV) and right atrium by color Doppler. Blood velocity in the right hepatic vein (HV) was recorded at its main stem 5 mm from the entrance into the inferior vena cava [9]. The flow in the ductus venosus (DV) was recorded at its most narrow part at origin from the portal sinus as described by Kiserud et al. [7], and umbilical venous blood velocity was recorded in its central intraabdominal between the DV and the abdominal wall [10]. The insonation angle was always kept below 30°. The venous blood velocity spectrum was analyzed for peak velocities at ventricular systole (S-velocity), additionally at end-systole (ES-velocity), at the diastolic peak (D-velocity), and during atrial contraction at the end of diastole (A-velocity) (Figure 1). Time-average maximum velocity was also evaluated. The blood velocity waveforms in the DV and HV were analyzed for pulsatility index for veins (PIVs) according to Hecher et al. [11], and S-A/S ratio as described by DeVore and Horenstein [12]. PIV values above the 95th percentile were defined as a sign of increased central venous pressure. A z-score as a measure of standard deviation from the gestational age mean for DV and HV PIVs was also calculated.

**Figure 2:**
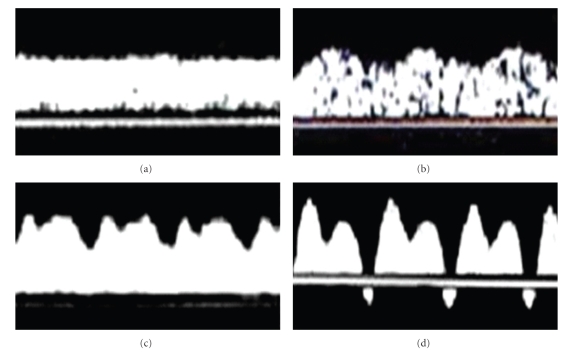
Umbilical venous blood velocity: (a) normal blood velocity, (b) flow with a single pulsation, (c) a double pulsation, and (d) triple-pulsating flow pattern.

Pulsating blood velocity in the umbilical vein was registered and defined as a decrease in velocity by more than 15% of the maximal velocity [10]. Pulsations corresponding to atrial contractions were defined as single and pulsations corresponding to both end-systole and to atrial contractions as double. The UV pulsation was defined as triple if the waveform was three-phase (Figure 2). All measurements were taken at fetal apnea and without fetal movements. In some of the cases the Doppler examination was repeated, but only the final examinations prior to birth, fetal demise, or termination of pregnancy were used for analysis. 

Furthermore, the results were related to reference data of venous Doppler for the hepatic vein [13] and ductus venosus blood velocity [11]. The data were also compared between groups with and without AV-valve insufficiency or umbilical venous pulsations, and between the different patho-physiological hydrops groups. Data were also correlated to perinatal outcome defined as survival, perinatal death, and termination of pregnancy in the different groups. The clinical management of the pregnancies relied only on the severity of the fetal hydrops, the presence of AV-valve regurgitation, and fetal behavior, but not on venous Doppler data. 

Fisher's exact test was used for comparison of frequencies and Wilcoxon signed-rank test for comparison of continuous variables. The Kruskal-Wallis nonparametric test was used to evaluate trends in z-score and UV groups. Level of significance was set at *P* < .05.

## 3. Results

All 100 fetuses had a moderate or severe hydrops. The average time interval between the last ultrasound examination and delivery, intrauterine death, or termination of pregnancy was 3 days (range 1–7 days). The mean gestational age at the last examination was 26 completed weeks and 5 days (range of 15 to 37 weeks). Fifty-six cases were examined once and 44 cases longitudinally.

The 100 cases were divided into groups based on the patho-physiological background. There were 32 in the high-output-heart-failure group, 24 in the low-output-heart-failure group, 25 in the group with hydrops caused by obstructed venous return, and 19 cases in the group with an unknown cause or chromosomal abnormality. Increased PIV (>95th percentile) in the DV was seen in 34 fetuses and 39 fetuses had increased HV PIV. Increased PIV was most frequent in the low-output hydrops group. 

There were 51 mortalities of which 27 were intrauterine deaths and 5 neonatal. Termination of pregnancy (TOP) was performed in 19 cases because of a progressing hydrops or severity of associated malformations. Five of these had chromosomal aberrations (4 Down's syndrome, 1 Turner syndrome), 7 had a congenital heart malformation, 1 had a mediastinal tumor, 1 had a lung malformation, 1 cholangiodysplasia, 2 had absent ductus venosus, and 1 had hydrothorax as the only indication for termination. TOP was also performed on parent request in one case. The relationship between cause of hydrops and mortality is given in Table 2. Mortality was most frequent in the low-output group (79%), which was mainly due to congenital heart malformation. Mortality was also high in the idiopathic group (68%), mainly in cases of chromosomal abnormalities (*n* = 7).

Gestational age at detection was similar for the different groups apart from the idiopathic group, which had chromosomal abnormalities that were terminated early. Gestational age was on average for that group 23 weeks (range 15–27); in the high-output-heart-failure group mean age was 26 weeks (range 20–26); in low-output-heart-failure group mean age was 28 weeks (range 21–34); and in case of venous obstruction mean age was 30 weeks (range 18–37).

DV and HV PIV z-scores did not differ between cases of mortality and survival. However, abnormal DV PIV was highly significantly related to mortality (*P* < .003). Abnormal HV PIV was also significantly related to mortality (*P* < .04). Mortality was significantly related to abnormal DV and HV PIV in the low-output heart failure (*P* < .01), but was not significantly related to venous blood flow in the other groups (Table 2).

**Figure 3:**
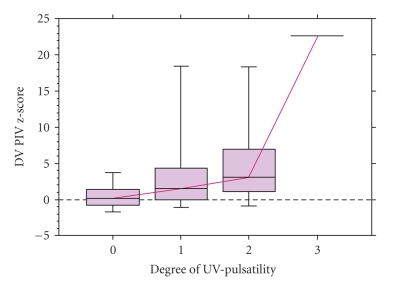
Umbilical venous pulsations in relationship with ductus venosus z-score of pulsatility index for veins. 0 = normal blood velocity; 1 = flow with a single pulsation; 2 = double pulsation; 3 = triple pulsating flow pattern.

Umbilical venous blood flow was recorded in all but one case. No pulsation was recorded in 49 fetuses and 50 had umbilical venous pulsations, that is, 26 with a single pulsation, 23 with a double pulsation, and one with a triple-phased pulsation. The relationship between UV pulsations and increased HV and DV PIV is given in Table 3 as well as mortality. There were 8 cases (16%) with no UV pulsations although the DV PIV was increased (PIV > 95th percentile). The DV PIV z-score and mortality increased with a higher degree of UV pulsatility (*P* < .0001, Figure 3, Table 2). All parameters of the DV waveform were related to UV pulsatility, but only the ES- and A-velocity in the HV blood velocity waveform. Reversal of DV flow in end-diastole was seen in 6 cases, 3 with single and 3 with double UV pulsations. Mortality was strongly related to the degree of UV-pulsatility (Table 3).

There were 31 fetuses with a normal venous Doppler recording in all 3 vessels. Survival in these cases was 65% (*n* = 20), and there was 11 mortalities (7 TOP) of which 3 had chromosomal abnormalities. The cause of hydrops was high-output heart failure in 11 (8 due to anemia), venous obstruction in 14 (5 with hydrothorax), and an idiopathic cause in 6 cases. 

Blood velocity over the tricuspid valve was recorded successfully in 96 fetuses and the mitral valve in 94. Tricuspid regurgitation (TR) was recorded in 34, of which 25 had holo- and 9 early systolic regurgitations. The main cause of TR was high-output heart failure due to anemia in Rh- incompatibility or parvovirus infection (*n* = 16). HV or DV PIV did not differ between cases with and without TR. These results also applied for different parts of the venous blood velocity waveform apart from ES and A blood velocity in the HV, which was significantly lower in the TR cases (*P* < .05). Mortality was not related to TR. There were 35 mortalities in the non-TR group (54.7%) and 16 in the TR group (47%). Mitral regurgitation (MR) was recorded in 13 fetuses, with 10 having a holosystolic MR. No correlation was found between MR and venous blood velocity.

## 4. Discussion

The results of the present study of pregnancies with fetal hydrops confirm previous reports on high mortality (50%). Normal venous blood velocity predicted a survival of 65%. Abnormal fetal venous Doppler was strongly related to mortality, even though there was a varying patho-physiological cause of fetal hydrops. These results apply for both the central fetal veins (HV, DV) and for the presence of umbilical venous pulsations. A strong correlation was seen between abnormal HV and DV blood velocity and UV pulsations, both in terms of frequency of abnormality and degree of blood flow velocity waveform abnormality. Venous Doppler can give information on prognosis and can therefore be recommended as a part of the routine workup of pregnancies complicated by fetal hydrops. 

Previously, the main cause of fetal hydrops was red blood cell incompatibility. Nonimmune fetal hydrops is presently more frequent after the introduction of immunoglobulin prophylaxis in at-risk mothers after delivery or an operative intervention during pregnancy. Different patho-physiological processes of varying etiology cause non-immune fetal hydrops. The prognosis is dependent on the underlying cause, associated fetal abnormalities, gestational age, and the presence and degree of fluid collection. Fetuses that develop non-immune hydrops later in gestation will have a lower mortality rate than those who develop it earlier. Antenatal diagnosis is important in order to predict the underlying cause and in some instances indicate management in order to improve survival, that is, on the fetus in-utero or the infant in the delivery room. 

Fetal systemic venous blood flow pulsates with a typical flow pattern directly related to increased central venous pressure [3, 4]. Increased central venous (right atrial) pressure will therefore alter the venous blood flow pattern with especially decreased diastolic blood flow velocities and often augment reversal of flow in end-diastole at the time of atrial contraction. Increased central venous pressure or hypoxia will facilitate opening of the ductus venosus and thus transmission of central venous pulsations into the umbilical vein. The pattern of UV pulsations will depend on the degree of DV opening [5, 13, 14]. Fetal arrhythmia can also cause alternation in fetal heart function and changes in central venous pressure and venous blood flow velocity [4]. A change in fetal heart rate might therefore be the cause of some of the abnormal blood velocity waveforms in the present study. However, increasing the upper cutoff for PIV to +3 SD did not change the results, neither for the HV nor for DV. 

As central venous blood flow is affected by atrial pressure, tricuspid regurgitation (TR) might change venous blood velocity. Leakage during systole might therefore increase atrial pressure and especially alter central venous blood flow in systole and early diastole. The present results showed only a correlation between TR and decrease in ES and A blood velocity in the HV. PIV in both the DV and HV was also higher in cases of TR. The lack of correlation between DV blood flow and TR might be that the main underlying cause was high-output heart failure in anemic fetuses.

UV pulsations and changes in diastolic blood velocities in the fetal inferior vena cava have been related to heart failure and mortality [6, 15]. These publications were based on cases of non-immune hydrops that were sent for echocardiographic evaluation. The relationship between abnormal venous blood velocity and mortality was stronger than in the present results, which might be due to larger etiological variation in the present study group. 

Cardiac function was mainly disturbed in fetuses with low-output cardiac failure due to severe cardiac defects such as structurally small hearts or obstruction of the great arteries (*n* = 13), and to arrhythmias (*n* = 3). Abnormal venous Doppler in the present study was most frequent in the low-output hydrops group. Abnormal DV PIV and UV-pulsations were seen in 18 cases (75%). The perinatal mortality rate was highest in this group (79%), which was mainly due to congenital heart malformations. The results are in agreement with Gembruch et al. [16] findings, suggesting that abnormal venous Doppler was related to myocardial dysfunction and severe right heart obstruction. Baez et al. [17] reported also an association between mortality and abnormal DV PIV, especially in congenital malformations affecting right ventricular function.

High-output cardiac failure was mainly caused by hypervolemia. Velocimetry in the precordial veins was only slightly affected. DV PIV was abnormal in only 8 cases (25%). The main cause of hydrops in the high-output group was fetal anemia (*n* = 22) due to both Rh-incompatibility and secondary to parvovirus infection. Only 2 of the 22 cases with hydrops due to anemia had abnormal DV PIV and UV-pulsations (9%). Long-lasting hypervolemia can lead to severe cardiomegaly, bilateral AV-valve regurgitation and finally cardiac failure similar to the findings of Hawkins et al. [18] in animal studies. However, the majority of the fetuses that had anemia could be treated successfully by intrauterine blood transfusions, which led to consecutive regression of the hydrops. Two fetuses with Rh-immunization developed intracerebral hemorrhage after transfusion in the 2nd trimester and died in-utero. Furthermore, two recipient twins and one fetus with DV agenesis died in-utero in congestive heart failure. 

Venous circulation was not significantly disturbed and the outcome was favorable in the majority of cases in the venous obstruction group (*n* = 25). Abnormal DV PIV was seen in 5 cases (20%) and 8 (32%) had UV pulsations. Fetal hydro- or chylothorax was the main cause of venous blood flow obstruction in this group (*n* = 14), 5 had a tumor in the thorax, and 4 had tumors in the abdominal cavity.

There were 19 cases included in the idiopathic group of hydrops. Cases of chromosomal abnormalities were included in this group due to uncertain cause of hydrops (*n* = 9); 5 of these were Turner syndromes. Consequently the mortality was high (68%). The mortality was 57% after exclusion of TOP cases. Abnormal HV PIV was seen in 6 cases in the idiopathic group (32%). The corresponding figure for the DV was 3 (16%) and UV-pulsations were registered in 10 cases (53%). 

The frequency of abnormality in venous blood velocity recording varied between vessels. Abnormal HV PIV was seen in 39 cases, but 34 had increased PIV in the DV. This difference might be explained by the HV being nearer to the heart. Changes in heart function might therefore be expressed earlier in the HV than in the DV. Pulsations in the UV were, however, more frequent (*n* = 49). There were 8 cases of increased DV PIV without UV pulsations. Fourteen fetuses had UV pulsations and normal DV PIV (4 with double pulsations and 10 with a single pulsation). In order to avoid overestimation of UV pulsation, a strict definition of UV pulsatility was applied: decrease in blood velocity during atrial contraction of more than 15% of the baseline maximum velocity. Furthermore, the recording was always performed in the central part of the umbilical vein in order to avoid possible pulsation when recordings were performed near the DV and the abdominal wall. There were no obvious patho-physiological groups represented in these pregnancies with normal DV flow and presence of UV pulsatility. Neither mortality nor type of cause differed in frequency from the rest of the pregnancies.

In summary, venous Doppler sonography of hydropic fetuses showed specific changes of the velocimetry in the HV, DV, and UV corresponding to changes in cardiac function that can be of value in predicting prognosis and outcome of pregnancy. Doppler velocimetry in the right HV, DV, and UV is a useful tool in the workup of hydropic fetuses.

## Figures and Tables

**Table 1:** Grouping of fetal hydrops by pathophysiological cause.

A. Hydrops caused by high-output heart failure (*n* = 32)

23 fetal anemia (13 Parvo B19 virus infection, 7 anti-D or anti Kell-immunization)
2 fetomaternal transfusion, 1 hemoglobulinopathy
3 twin-twin transfusion syndrome
1 large sacrococcygeal teratoma
5 atypical course of the umbilical vein

B. Hydrops caused by low-output cardiac failure (*n* = 24)

10 severe obstruction of the cardiac outflow tract
(1 HRHS, 2 PS+TR, 4 HLHS, 2 AoS+ MR, and 1 CoAo)
5 small heart syndromes (size of 17 to 19 mm)
4 arrhythmia (3 SVT, 1 AV-block 30)
2 myocardial hypertrophy
2 rhabdomyoma
1 atrial aneurysm

C. Hydrops caused by obstruction of venous return (*n* = 25)

13 uni- or bilateral hydrothorax
4 lung malformation (3 CCAML, 1 sequestration)
3 tumor in the mediastinum or in the liver
2 meconiumileus
1 megacystis
1 cholangiodysplasia
1 diaphragmatic hernia

D. Hydrops of idiopathic cause (*n* = 19)

9 hydrops with unclear or multiple diagnosis
10 chromosomal aberrations
(5 trisomy 21, 3 Turner Syndrome, 1 triploidy, 1 marker chromosome)

HRHS: hypoplastic right heart syndrome; PS: pulmonary stenosis; TR: tricuspid regurgitation;

HLHS: hypoplastic left heart syndrome; AoS: aortic stenosis; MR: mitral regurgitation; CoAo: coarctation of the aorta; SVT: supraventricle tachycardia.

**Table 2:** Relationship between cause of hydrops and mortality and abnormal venous blood velocity.

	Number	Mortality	Mortality minus termination	Abnormal HV PIV	Abnormal DV PIV	UV pulsations
Number		51	32	39	34	49
High-output heart failure	32	9 (28%)	7 (23%)	11 (34%)	8 (25%)	13 (41%)
Low-output heart failure	24	19 (79%)	10 (67%)	15 (63%)*	18 (75%)*	18 (75%)*
Venous obstruction	25	10 (40%)	7 (32%)	7 (28%)	5 (20%)	8 (32%)
Idiopathic	19	13 (68%)	8 (57%)	6 (32%)	3 (16%)	10 (53%)

DV: ductus venosus; HV: right hepatic vein;

PIVs: pulsatility index for veins; UV: umbilical venous.

Abnormal PIV was defined as > 95th percentile.

* = *P* < .01.

**Table 3:** Type of umbilical venous pulsations (UV) in relationship to increased pulsatility index for veins (PIV) in the right hepatic vein (HV) and ductus venosus (DV) and mortality. Numbers and percent are given.

UV pulsation	*n*	Increased HV PIV	%	Increased DV PIV	%	Mortality	%	Mortality minus termination	%
None	49	11	22	8	16	19	39	14	33
Single	26	15	58	11	42	12	46	6	33
Double	23	12	52	14	61	18	78	11	69
Triple	1	1	100	1	100	1	100	0	0
